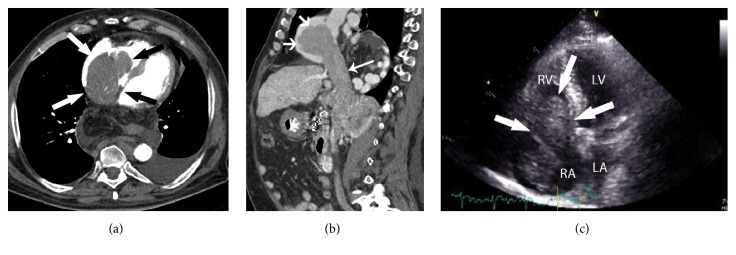# Vena Cava Superior Syndrome Related to Right Heart Invasion of an Unresectable Hepatocellular Carcinoma

**DOI:** 10.1155/2016/6985917

**Published:** 2016-03-20

**Authors:** Geert Maleux, Werner Budts, Vincent Vandecaveye

**Affiliations:** ^1^Department of Radiology, University Hospitals Leuven, Herestraat 49, 3000 Leuven, Belgium; ^2^Department of Cardiology, University Hospitals Leuven, Herestraat 49, 3000 Leuven, Belgium

## Abstract

A rare case of hepatocellular tumor extension in the right heart was reported. The patient presented with clinical signs of a vena cava superior syndrome. Computed tomography and transthoracic echocardiography demonstrated a large tumoral mass lesion extending from the left liver lobe into the inferior vena cava right atrium and right ventricle. The patient was treated with best supportive care.

## 1. Case Presentation

A 75-year-old man with a history of alcoholic liver cirrhosis and hepatocellular carcinoma presented with a deterioration of his general condition, associated with clinical signs of vena cava superior syndrome: swollen and painful face and dilated subcutaneous jugular veins. The carcinoma was diagnosed earlier by cross-sectional imaging, confirmed by laboratory analysis and biopsy, and already consecutively treated for 8 years by, respectively, surgical right hepatectomy, radio-frequency ablation, chemoembolization with and without drug-eluting beads, and catheter-directed yttrium-90 radioembolization. Axial (Figure 1(a)) and reconstructed sagittal (Figure 1(b)) contrast-enhanced computed tomography (CT) images revealed a large, enhancing tumor mass lesion (large arrows), originating from the left liver lobe and extending into the right atrium up to the right ventricle; extension of the tumor was also identified into the inferior vena cava (small arrow). Transthoracic echocardiography (Figure 1(c)) confirmed a slightly hyperdense mass lesion (white arrows) protruding into the right atrium (RA) and right ventricle (RV); the left atrium (LA) and left ventricle (LV) were both tumor-free. Based on these 2 independent imaging modalities, it was clear that the hepatocellular carcinoma grew not only from the left liver lobe into the inferior vena cava, but also into the right atrium and ventricle. It thereby obliterated nearly completely the right heart cavities, obstructed the drainage of vena cava superior, and was subsequently also associated with a vena cava superior syndrome.

## 2. Discussion

Hepatocellular carcinoma-related venous and atrial involvement is rare and mainly occurs as end-stage disease [1]. Clinical symptoms are mainly swollen lower extremities and Budd-Chiari syndrome, including ascites, abdominal pain, and hepatomegaly. Vena cava superior syndrome might occur and is mostly associated with widespread hepatocellular carcinoma metastatic to mediastinal lymph nodes, thereby narrowing or occluding the superior vena cava [2]. Treatment options are very limited and include radical surgical resection in very rare cases [3], palliative interventional stent implantation [2], or more frequently best supportive, palliative care. In this case it was obvious that surgery was not an option. Transatrial stent placement, potentially effective in cases of cavoatrial involvement, would not have been effective as well, as in this case the tumor extended into the right ventricle. Therefore, the patient was treated with best supportive care and died a few weeks later.

In conclusion, a rare case of hepatocellular tumor extension in the right heart with vena cava superior syndrome as end stage disease was clearly documented by CT and transthoracic echocardiography.

## Figures and Tables

**Figure 1:**